# How an Infection of Sheep Revealed Prion Mechanisms in Alzheimer’s Disease and Other Neurodegenerative Disorders

**DOI:** 10.3390/ijms22094861

**Published:** 2021-05-04

**Authors:** George A. Carlson, Stanley B. Prusiner

**Affiliations:** 1 Institute for Neurodegenerative Diseases, Weill Institute for Neurosciences, University of California San Francisco, San Francisco, CA 94158, USA; george.carlson@ucsf.edu; 2 Department of Neurology, Weill Institute for Neurosciences, University of California San Francisco, San Francisco, CA 94158, USA; 3 Department of Biochemistry and Biophysics, University of California San Francisco, San Francisco, CA 94158, USA

**Keywords:** Aβ, α-synuclein, Alzheimer’s disease, neurodegenerative disease, prions, PrP, tau

## Abstract

Although it is not yet universally accepted that all neurodegenerative diseases (NDs) are prion disorders, there is little disagreement that Alzheimer’s disease (AD), Parkinson’s disease, frontotemporal dementia (FTD), and other NDs are a consequence of protein misfolding, aggregation, and spread. This widely accepted perspective arose from the prion hypothesis, which resulted from investigations on scrapie, a common transmissible disease of sheep and goats. The prion hypothesis argued that the causative infectious agent of scrapie was a novel proteinaceous pathogen devoid of functional nucleic acids and distinct from viruses, viroids, and bacteria. At the time, it seemed impossible that an infectious agent like the one causing scrapie could replicate and exist as diverse microbiological strains without nucleic acids. However, aggregates of a misfolded host-encoded protein, designated the prion protein (PrP), were shown to be the cause of scrapie as well as Creutzfeldt–Jakob disease (CJD) and Gerstmann–Sträussler–Scheinker syndrome (GSS), which are similar NDs in humans. This review discusses historical research on diseases caused by PrP misfolding, emphasizing principles of pathogenesis that were later found to be core features of other NDs. For example, the discovery that familial prion diseases can be caused by mutations in PrP was important for understanding prion replication and disease susceptibility not only for rare PrP diseases but also for far more common NDs involving other proteins. We compare diseases caused by misfolding and aggregation of APP-derived Aβ peptides, tau, and α-synuclein with PrP prion disorders and argue for the classification of NDs caused by misfolding of these proteins as prion diseases. Deciphering the molecular pathogenesis of NDs as prion-mediated has provided new approaches for finding therapies for these intractable, invariably fatal disorders and has revolutionized the field.

## 1. Introduction

Prions are composed of host-encoded proteins that adopt alternative conformations, which are self-propagating [1,2,3,4]. PrP prion diseases, including “mad cow” disease, which was epidemic in the United Kingdom in the 1980s and 1990s, were thought to be the only infectious neurodegenerative diseases (NDs) [5,6]. However, evidence has shown that Aβ amyloid pathology characteristic of Alzheimer’s disease (AD) has been transmitted to patients by inoculation of human growth hormone that was contaminated with Aβ amyloid [7,8]. Transmission of misfolded Aβ aggregates (i.e., prions) has been demonstrated in experimental animals models and in cell cultures [6,9,10,11,12]. Additionally, tauopathy from patients with AD was transmitted to human tau transgenic mice, arguing that tau prions can induce conformations in wild-type or mutant tau protein that initiate prion propagation [13]. Similarly, α-synuclein prions that cause multiple system atrophy (MSA) can transmit disease to transgenic mice and infect cultured reporter cells [14,15]. The transmission of pathology from diseases involving Aβ, tau, or α-synuclein misfolding in these (and other) NDs argues that the approaches used to unravel the mystery of scrapie, the prototypical PrP prion disease, can be successfully applied to understanding other NDs. The shared feature of transmissibility, along with other properties discussed below, suggest that prion diseases can involve proteins other than PrP, just as there are a variety of viral, bacterial, and rickettsial disorders.

## 2. Scrapie and the Prion Protein

Scrapie, an ND of sheep and goats, has been known for more than 200 years. The condition causes animals to itch incessantly (“scraping” against fences with subsequent loss of fleece) and is accompanied by loss of appetite, emaciation, and death. By the 18th century, and likely earlier, the agricultural community in Europe knew that scrapie was contagious [16]. When shepherds identified a member of their flock showing signs of the disease, they would isolate it from the healthy animals. Transmission by inoculation was established during the first half of the 20th century [17,18]. This finding led to a microbiological perspective in studies on scrapie, and later on related human diseases, as well as a focus on isolating and characterizing the peculiar infectious agent that caused spongiform degeneration of the brain. It was entirely logical for scientists to search for a nucleic acid core in the scrapie agent. Tikvah Alper and her colleagues, however, demonstrated that the scrapie agent was extremely resistant to inactivation by ultraviolet light and ionizing radiation and estimated the size of the infectious particle to be about 90% smaller than the smallest known virus [19,20,21]. This led to the proposition that the scrapie agent lacked a functional nucleic acid [20]. Not unexpectedly, this proposal was widely rejected, but it was ultimately demonstrated to be true by the discovery of the prion and its unprecedented mechanisms of replication and pathogenesis [2,22]. A prion was originally defined as “a small proteinaceous infectious particle which is resistant to inactivation by most procedures that modify nucleic acids” [1]. As more has been learned, a refined definition has been proposed: “Prions are composed of host-encoded proteins that adopt alternative conformations, which are self-propagating” [23].

As scientists were trying to identify the scrapie agent, a novel neurological disease called kuru was described among the Fore tribe in remote Papua New Guinea [24,25]. The affected individuals presented with headache and joint pain that rapidly progressed to ataxia in 6 to 12 weeks and to death within 12 months. Histopathologic similarities between kuru and scrapie were noted by Hadlow, who proposed an infectious etiology for kuru similar to scrapie based on indistinguishable neuropathologies [26]. Gajdusek and coworkers inoculated kuru brain homogenates into the brains of non-human primates that developed progressive neurological dysfunction and pathology similar to kuru within three years [27]. The transmission of kuru within the Fore tribe was attributed to ritualistic cannibalism during funeral rites. Like scrapie, kuru had very long asymptomatic incubation periods. Inoculation of scrapie brain homogenates produced incubation periods as long as 30 months in sheep and goats, whereas epidemiological studies of kuru in the Fore tribe suggested incubation periods several decades long.

A long asymptomatic period that precedes disease onset is a feature of many NDs that is shared with scrapie and kuru. Only rarely are young people afflicted with neurodegenerative prion diseases. However, AD pathologies are seen in cognitively normal, middle-aged individuals and in young adults with familial AD (fAD) mutations well before symptoms appear [28,29]. This is similar to experimental scrapie in which accumulation of PrP^Sc^ is apparent well before clinical signs are evident. Transgenic models of MSA and frontotemporal dementia (FTD) show accumulation of pathological aggregates of α-synuclein and tau before clinical disease is apparent. Therefore, long asymptomatic periods concurrent with pathological accumulation of misfolded protein is a feature common to neurodegenerative prion diseases.

The pathogens causing scrapie, kuru, and CJD had other unusual properties in addition to their prolonged incubation periods and resistance to inactivation by irradiation. The prions causing each disorder were extremely resistant to inactivation by formalin fixation or heat [30,31] and failed to elicit an immune response during infection. Notably, an immune response to foreign pathogens is common in infected animals [32]. These strange pathogens were often dubbed “slow viruses” and later “unconventional viruses” [33,34]. The speculation that the infectious agent causing scrapie might lack a nucleic acid genome [20] generated several hypotheses that proposed novel mechanisms for the reproduction of these unprecedented agents [35,36].

To identify the composition of the infectious scrapie agent, one of us (S.B.P.) used biochemical purification based on enriching for infectivity as assessed by the incubation time in hamsters. Each fraction from the enrichment process, which included a limited proteinase K digestion step, was inoculated intracerebrally into groups of hamsters to determine the incubation time. Use of incubation time (rather than traditional end point titration) to estimate the titer of infectivity was a major advance. These highly purified fractions retained significant infectivity in the animals, and the infectious protein was identified and named PrP for “prion protein” [1,37].

Interestingly, the mRNA that encodes PrP was also identified in uninfected hamsters; however, the PrP produced in uninfected animals was found to be susceptible to limited proteolysis, denoted PrP^C^, whereas the disease-specific form was protease-resistant, designated PrP^Sc^ [38]. It became clear that the amino acid sequences of PrP^C^ and PrP^Sc^ were identical after the discovery that both isoforms were encoded by the same gene.

## 3. Prion Protein and Scrapie Incubation Time Genes Are Linked

The DNA sequence of the mouse PrP gene (*Prnp*) provided a new tool to re-evaluate earlier genetic studies on the mouse-adapted scrapie agent. Two widely available inbred strains of mice that differed widely in incubation times after inoculation with the Rocky Mountain Lab (RML) prion strain were used to test genetic linkage of this key scrapie phenotype to the PrP gene. After inoculation with the RML strain of scrapie prions, I/LnJ strain mice had very long incubation periods (255 ± 14 days (SEM)), whereas NZW/LacJ mice had much shorter ones (113 ± 2.8 days). (NZW × I/Ln)F1 hybrid mice had incubation times of 223 ± 2.8 days, indicating that longer incubation times were dominant. Incubation periods in the offspring of a backcross of (NZW × I/Ln)F1 mice (long incubation time) to NZW mice (short incubation time) segregated into two groups, demonstrating that a single Mendelian gene controlled the incubation time [39]. The NZW strain and most other inbred mice carry the *‘a’* allele of the gene encoding PrP (*Prnp*), which was originally defined by differences in a restriction enzyme fragment length polymorphism that distinguished it from the less common *Prnp^b^* allele [39,40]. The discovery of genetic linkage between scrapie incubation time and *Prnp* provided the first unbiased biological evidence supporting the relevance of the biochemical association of PrP^Sc^ with the prion particle. The *a* and *b* alleles of *Prnp* were found to differ at codons 108 (Leu/Phe) and 189 (Thr/Val) [41]. This coding difference raised the possibility that *Prnp* itself controlled the incubation time. Genetic recombination events that separated an incubation time from *Prnp* could not be identified despite considerable effort [40,42]. This was not formally proven, however, until 1998 when one *Prnp* allele was converted to the other by knock-in technology [43].

## 4. Scrapie Strains

Well before the discovery of PrP, Dickinson and Mackay reported scrapie incubation times that ranged from ~120 to nearly 300 days in various inbred mouse strains that were inoculated with the identical scrapie brain homogenate [44]. Backcrosses and intercrosses involving mouse strains with short incubation times and long incubation times demonstrated that a single autosomal gene, named *Sinc* for scrapie incubation, was responsible for most of the variance among inbred mouse strains. *Sinc* had two alleles (*p7* for prolonged and *s7* for short ME7 strain incubation times). Only two closely related mouse strains in the United Kingdom, VM/Dk and IM/Dk, carried the *p7* allele [40]. Importantly, long and short incubation times were not stable phenotypes of each of the two *Sinc* alleles but reflected interactions between host genotype and the particular strain of scrapie agent. For example, VM and IM mice homozygous for the “long incubation time” *Sinc^p7^* allele had much shorter incubation times for some scrapie strains, such as 87V, than did mice with the “short incubation time” allele—the opposite of the results obtained with the ME7 strain [45]. Many distinct strains of the presumed scrapie virus were defined according to their incubation times, the distribution of pathological lesions, and the clinical signs in *Sinc^s7^*, *Sinc^p7^,* and *Sinc* heterozygous F1 mice [46,47]. Unfortunately, the scrapie prion strains and the *Sinc^p7^* mice that defined them were not widely available. This greatly delayed determining whether *Sinc* and the *Prnp*-linked incubation time were the same gene. For decades, the very existence of distinct microbiological strains provided the strongest and most compelling argument against an infectious agent devoid of functional nucleic acid—because what other than nucleic acid could encode heritable information and be subject to mutation and selection [45,46,47]?

Because PrP^Sc^ is a functional component of the scrapie prion, infectivity produced by *Prnp^a^* mice would be composed of PrP^Sc^-A molecules with a different amino acid sequence from PrP^Sc^-B made in *Prnp^b^* mice. Thus, there was no need to invoke nucleic acid to account for different prion properties resulting from passage between strains that differed at *Prnp*. For example, mice expressing PrP-B had longer incubation times when inoculated with RML prions that were maintained in mice expressing PrP-A than with RML prions that had been passed through PrP-B mice, suggesting a barrier to efficient prion transmission [48]. Although passage of RML prions through PrP-B mice dramatically shortened the incubation time in these mice, incubation times in PrP-A mice were always shorter than in PrP-B mice regardless of passage history, indicating that something in addition to the PrP amino acid sequence was necessary to account for the properties of some prion strains.

Because *Prnp^b^* behaves as a dominant allele in prolonging incubation time of RML prion isolates, it was expected that expression of *Prnp^b^* transgenes in *Prnp^a^* mice would increase incubation times [49], but incubation times of mice expressing *Prnp^b^* transgenes were shortened. This shortening of incubation time was actually due to the dramatically increased amount of transgene-encoded PrP-B substrate, which, though less efficiently converted to PrP^Sc^ than PrP-A, still supports prion replication and causes disease. Thus, the dominance of *Prnp^b^* in *Prnp^a^/Prnp^b^* heterozygous mice reflects the reduced supply of PrP^C^-A produced from the single copy of *Prnp^a^* rather than an inhibitory effect of PrP-B on prion replication [50].

**Figure 1:**
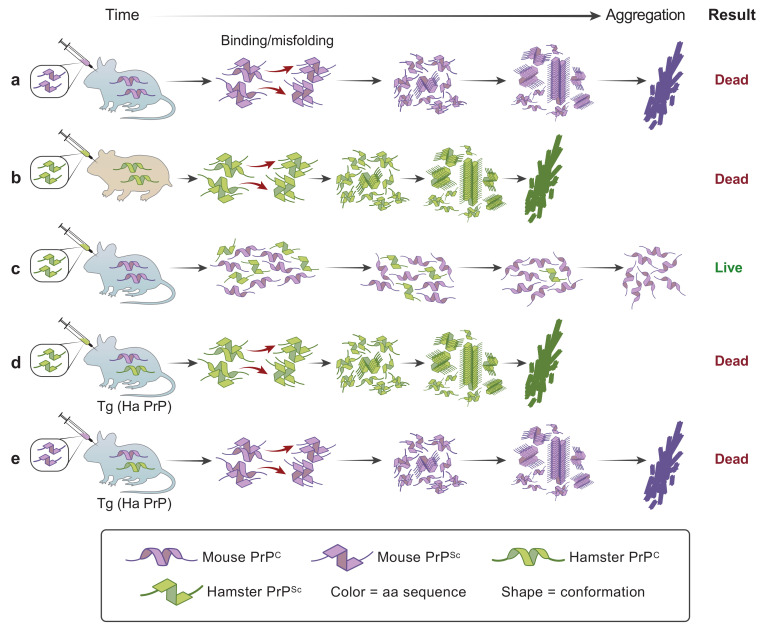
PrP is responsible for the species barrier of mice to infection with hamster prions. Color is used to indicate differences in the amino acid sequence, with purple indicating mouse PrP and green indicating hamster PrP molecules. PrP^C^ and PrP^Sc^ conformations are indicated by the shape of the icons, with helical shapes indicating PrP^C^ and angular shapes illustrating misfolded PrP^Sc^ conformations. Mouse prions efficiently bind and misfold mouse PrP (**a**), and hamster prions efficiently bind and misfold hamster PrP (**b**). However, hamster PrP^Sc^ fails to bind and cause misfolding of mouse PrP^C^ with subsequent aggregation and death of the animal (**c**). Mice expressing hamster PrP transgenes (indicated by “Tg”) are susceptible to hamster prions, and the prions that are produced are encoded by the hamster (Ha) PrP transgenes. Because Tg(HaPrP) mice also express endogenous mouse PrP, they also are susceptible to mouse prions, producing mouse, but not hamster PrP^Sc^ (**d**,**e**).

**Figure 2:**
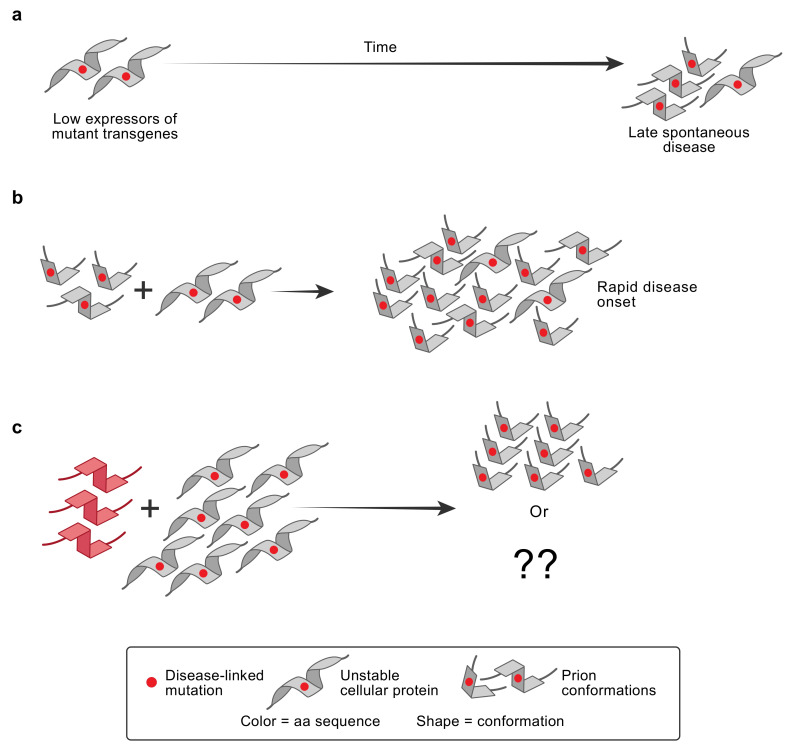
Use of high-level expression of transgene-encoded disease-linked protein to accelerate ND research. Smooth gray helical icons indicate the cellular form of the ND protein, with a disease-linked mutation indicated by a small red circle. Angular icons indicate misfolded prion conformations. Mice expressing a low level of transgene-encoded disease-linked mutant protein develop spontaneous disease only very late in life (**a**); however, these unstable mutant proteins provide a substrate for the replication of homologous prions (**b**) or, potentially, mismatched prions (red) that can template propagation of their own conformation or, possibly, give rise to new prion conformations, as shown in the reaction in (**c**). Note that prions can adopt more than one conformation in an individual.

In general, prions with the same PrP amino acid sequence as the host have shorter incubation times than mismatched prions. The species barrier to prion transmission provides an excellent example [51,52,53,54]. Syrian-hamster-adapted scrapie prions have incubation times ranging from ~60 to 150 days in hamsters. Inoculation of hamster prions into mice produced incubation times longer than 300 days, and in some cases, the mice did not show clinical signs at all. However, inoculation of brain homogenates from the mice that did become ill following inoculation with hamster prions caused disease in mice with incubation times that were similar to those produced by mouse scrapie prions. Transgenesis provided the tool to demonstrate that the species barrier between hamsters and mice was due solely to PrP. Lines of mice were produced that expressed both endogenous mouse PrP and transgene-encoded Syrian hamster PrP (SHaPrP), whose amino acid sequences differ by ~10%. When these mice were inoculated with mouse PrP^Sc^ prions, only mouse PrP^C^ was converted to PrP^Sc^, whereas when inoculated with hamster PrP^Sc^ prions, only hamster PrP^C^ was converted into PrP^Sc^ [54]. The dependence of the species barrier on PrP is illustrated in Figure 1. The distribution of pathological lesions and the presence of PrP amyloid plaques in the transgenic mice also were very similar to scrapie in hamsters. This study revealed the greater efficiency of homotypic interactions between PrP^C^ and PrP^Sc^ for the replication of prions. Based on these and other findings, prion replication appeared to occur through binding of PrP^Sc^ to PrP^C^, which induced conversion of PrP^C^ to PrP^Sc^, thereby increasing the amount of the infectious agent. Mice expressing SHaPrP transgenes had SHa prion incubation times as short as 50 days in high-copy-number transgenics. Incubation times were inversely proportional to transgene copy number and the level of SHaPrP [53,54]. The discovery that high levels of PrP expression dramatically accelerated disease onset and rate of progression has had far reaching impacts on the use of transgenic mice to study PrP prions and other NDs, as illustrated in Figure 2. Further acceleration of prion disease was obtained by overexpression of prion transgenes with disease-linked mutations that render the cellular form of the prion unstable and more readily converted to misfolded prion conformations, as discussed below.

## 5. *PRNP* and Familial Prion Diseases

The discovery that allelic forms of *Prnp* controlled mouse scrapie incubation times led to studies to test whether rare familial forms of GSS were also influenced by the nucleic acid sequence of the human PrP gene, *PRNP*. A missense mutation at codon 102 was linked to GSS [55], and transgenic mice that expressed high levels of *Prnp* with the mouse equivalent of the human GSS mutation spontaneously developed an ND that could be transmitted with short incubation times to transgenic mice that expressed GSS-mutant PrP at low levels and that developed spontaneous disease only late in life [56,57]. Figure 2 illustrates the now widespread use of transgenic mice expressing low levels of disease-linked mutant transgenes to study misfolding, based on the approach pioneered with GSS PrP prions.

An important feature of PrP prion diseases is that they can have infectious, sporadic, or familial origins [2,22]. Infectious and iatrogenic PrP prion diseases, exemplified by kuru and CJD caused by prion-contaminated human growth hormone [58], are exceptionally rare. Sporadic prion disease is the most common form, and less than 20% of prion disease cases have been reported to be associated with mutations in *PRNP*. It is likely that the actual number of causative *PRNP* mutations is extremely low. Although a variety of *PRNP* mutations have been reported, many of these were not identified in multigenerational families with genetic linkage data. Genetic linkage provides strong evidence that some *PRNP* mutations, exemplified by GSS-linked P102L, are causative and fully penetrant [55,59,60]. Although more than 60 mutations in the *PRNP* gene have been reported to be pathogenic, Minikel and co-workers [61] used large-scale population data to assess the probability of individual *PRNP* variants being causative. Many *PRNP* mutations that had previously been thought to cause disease were found in the control population but were rarely, if ever, identified in patients with confirmed PrP prion disease and are thus very unlikely to cause PrP prion disease.

**Figure 3:**
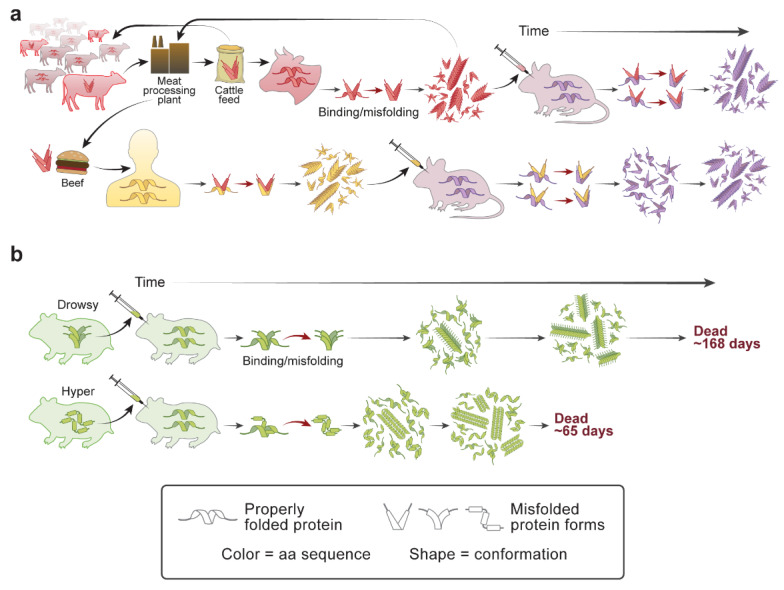
The same or similar misfolded prion conformations can be adopted by proteins that differ in amino acid sequence. (**a**) Reactions illustrate transmission of BSE, which was a consequence of industrial cannibalism when misfolded bovine PrP^Sc^ (red angular icons) were incorporated during meat processing and included in cattle feed resulting in the BSE epidemic. Meat or beef by-products for human consumption were also contaminated with BSE prions, resulting in variant (v) CJD (human PrP, yellow angular icons). Both BSE and vCJD could be transmitted by inoculation into mice (purple icons) or, by ingestion of BSE, into humans (yellow icons). These results suggest that bovine, human, and mouse PrP, although they differ in primary structure, can misfold into conformations that produce similar clinical signs. (**b**) The illustration shows transmission of hyper and drowsy strains of hamster prions that share the same PrP amino acid sequence but have different conformations and clinical signs.

The propensity of PrP variants for misfolding and aggregation is experimentally tractable both in culture and in mouse models, but how a particular primary structure of PrP affects conformation cannot be predicted at present. In general, mismatching of PrP^Sc^ and PrP^C^ amino acid sequences results in inefficient disease transmission, but prediction of whether a particular prion will cross a species barrier has not been possible, to date. For example, bovine spongiform encephalopathy (BSE) prions can infect humans, albeit at low efficiency, and cause a variant form of CJD (vCJD) despite differences in human and bovine amino acid sequences. Figure 3a illustrates the effects of conformation versus sequence on prion transmission and strain formation. Despite differences in their primary structures, both vCJD and BSE can also be transmitted to mice, and evidence supports the hypothesis that these diseases share the same or similar prion strain conformations. The two isolates produce species-analogous profiles of pathology and patterns of PrP^Sc^ deposition within the brain.

## 6. Protein Conformation Enciphers Heritable Information

Experimental transmission of transmissible mink encephalopathy (TME), a PrP prion disease, to Syrian hamsters resulted in two distinct strains of prions, designated hyper (HY) and drowsy (DY), which were stable through repeated passage [62]. The strains differed in incubation time, distribution of pathological lesions within the brain, and clinical signs due to the different conformation of PrP in the two strains (Figure 3b). The amino acid sequences of PrP in the HY and DY strains are identical, but PrP^Sc^ differs in the cleavage sites of proteinase-K (PK) digestion due to conformational differences between the two prion strains [63,64].

Similar evidence that the heritable prion strain information is encoded by protein conformation comes from transmission of two distinct human prion diseases into mice [65]. Evidence argues that conformation alone encrypts consistent heritable properties of different strains passaged in the same inbred strain of mice, a finding that had previously been attributed to nucleic acid genomes [65,66,67]. The importance of conformation in dictating prion properties is reinforced by the existence of an extremely rare sporadic form of fatal insomnia (sFI) that lacks any mutation in *PRNP* [68].

## 7. Genetic Control of Susceptibility to Human PrP Prion Diseases

Epidemiological evidence demonstrated that *PRNP* polymorphisms influence susceptibility to kuru, similar to the genetic control of scrapie incubation period in mice. Heterozygosity at *PRNP* codon 129 was associated with longer incubation times and survival following consumption of tissue infected with kuru prions among the Fore tribe. The tribe ceased ritual cannibalism in the 1950s, and incubation times exceeding 50 years have been documented [69]. Virtually all kuru patients who lived to old age or failed to develop disease despite exposure were heterozygous at codon 129, whereas those afflicted at a young age (putative short incubation times) were homozygous for the M or V allele. Thus, kuru imposed a strong balancing selection on the Fore that essentially eliminated codon 129 homozygotes from the population [70].

Genotyping Papua New Guineans also revealed a novel allele of *PRNP* (G127V) that was common within the kuru region but not found in any individuals with kuru [70]. Thus, *PRNP* V127, which was always inherited with M129, appeared to be a resistance allele that was recently selected during the kuru epidemic; its presence was postulated to confer complete resistance to kuru. Testing susceptibility in transgenic mice confirmed the validity of this observation [71]. The effects of V127 and all other variants of PrP that affect disease susceptibility, incubation time, clinical signs, or distribution of pathological lesions most likely are mediated by their effects on PrP conformation.

The fact that PrP^C^ and PrP^Sc^ are encoded by the same amino acid sequence indicates that protein sequence does not dictate a single conformation. It is also likely that multiple conformations of PrP^Sc^ exist in a single infected animal. Properties of prion strains can change when passaged in different cell lines, when going from mice to cell culture (and vice versa), or in the presence of drugs that prolong scrapie incubation time in mice [72,73,74]. Evolution of prion strains and acquisition of drug resistance either by induced conformational change or selection of preexisting conformers has important implications for the development of prion therapeutics. Particularly relevant is the failure of drugs that dramatically prolong mouse prion incubation times to have any effect on replication of CJD prions in humanized mice [73].

Spread of misfolded protein aggregates along neural pathways is a feature of animal models of NDs such as Parkinson’s disease (PD) and AD, which are much more common than PrP prion diseases [10,29,75]. Pathology can also be transmitted by inoculating mice or cell cultures with aggregates of tau, Aβ, or α-synuclein [14,15,76,77,78]. PrP is not the only protein driving neurodegeneration that can produce multiple conformers from a single primary structure [79,80]. As only one example, Aβ transmission can lead to distinct strains of Aβ prions [81]. Multiple conformations of misfolded proteins subject to selective pressures from drugs or host resistance can lead to the emergence of “mutant” forms with different properties. This phenomenon presents a challenge even more difficult than that in cancer therapy in which malignant cells evolve through the selection of mutations in the cellular DNA. In cancer, identifying the genes involved in resistance to therapy offers new potential targets and pathways. In contrast, NDs involve a fluid set of protein conformations that, at present, cannot be predicted from the amino acid sequence, which at least partially explains the lack of any effective therapies for prion diseases caused by PrP, Aβ, tau, α-synuclein, and other neurodegenerative prion diseases.

## 8. Prion Replication of Misfolded Proteins Causing AD, PD, and MSA

Studies of prion diseases caused by aggregates of misfolded PrP pointed to the likelihood that other NDs were caused by prion forms of other proteins. The tools and approaches that were successfully used to discover PrP and a new principle of pathogenesis were applied to other NDs, and the results were consistent with the prion mechanism. One reason for the success of studies with scrapie was that PrP^Sc^ is infectious and, in addition to its spread from cell to cell along neural pathways, could be transmitted experimentally by inoculation. A common argument against prions and prion mechanisms being involved in a variety of NDs is that these NDs are not contagious. However, misfolded proteins from non-PrP NDs readily infect reporter cells in culture and spread from infected cells in the brain to non-infected cells. Thus, we use nomenclature to name prions according to the particular protein that misfolds and aggregates—that is, there are Aβ prions, tau prions, α-synuclein prions, and many others (Table 1).

## 9. Aβ Prions

AD is the most common ND and presents as a gradual decline in cognitive ability. The key histopathological hallmarks of AD are Aβ amyloid plaques and neurofibrillary tangles (NFTs) and loss of neurons and synapses [82]. In cerebral amyloid angiopathy (CAA), a different Aβ prion disease, Aβ amyloid fibrils accumulate in and around small blood vessels in the brain, but with no substantial tau accumulation or formation of NFTs [83,84].

The Aβ peptide is derived from the amyloid precursor protein (APP) by endoproteolytic cleavage [85,86]. Though most AD is sporadic, the small number of fAD cases provides insight into the disease. Mutations in the *APP* gene, but outside the Aβ-peptide sequence itself, lead to an elevation of Aβ peptide levels. Thus, the DNA sequence of the Aβ peptide itself is identical in healthy individuals and AD patients. In addition to mutations in the *APP* gene, many mutations occur in the *PSEN1* and *PSEN2* genes, whose proteins are responsible for the processing of APP. These mutations increase production of a variety of Aβ peptides, with Aβ40 and Aβ42 peptide isoforms predominating in AD [87,88,89]. Studies have demonstrated that Aβ42 is the most aggregation-prone and amyloidogenic of these peptides and the major species that accumulates in Aβ plaques in AD.

After demonstrating the transmissibility of kuru and CJD, Gajdusek and Gibbs applied the same hypothesis to the transmissibility of AD [90,91]. Brain homogenates from 52 AD cases were injected into non-human primates, and animals injected with two fAD cases developed a disease that was indistinguishable from CJD [90]. These results, however, could not be replicated, given the possibility of contaminating PrP prions. In similar experiments, marmosets were inoculated with AD brain homogenates, and after almost 10 years of incubation, the majority of the animals had amyloid plaques that stained positive with anti-Aβ antibodies [9,92]. These investigations provided evidence for the transmissibility of Aβ pathology; however, they were difficult given the long incubation periods and high cost of housing nonhuman primates.

Later, Jucker, Staufenbiel, and Walker created transgenic mice expressing mutant APP [93]. These mice were injected with AD brain homogenates both intracerebrally and intraperitoneally, and both routes were shown to accelerate the deposition of amyloid plaques. This experimental design was used in earlier experiments demonstrating the transmissibility of the PrP prion disease GSS [94,95]. Intracerebrally injecting synthetic mutant Aβ fibrils into other transgenic mouse lines also induced Aβ plaque deposition, and depending on the APP mutation, the site of proteolytic cleavage was modified, resulting in amyloid fibrils with different characteristics [81,96]. Transgenic mice expressing low levels of GSS-mutant PrP developed disease only late in life, if at all. Inoculating these low expressors with brain homogenates from mice that developed spontaneous disease dramatically accelerated disease. These studies set the precedent for the now common use of transgenic mice expressing low levels of mutant transgenes as assays for prions of various types [97].

Iatrogenic cases of PrP prion diseases (iCJD), which are rare, have occurred via medical procedures using cadaveric human growth hormone, dura mater grafts, or corneal transplants from patients that had accumulated PrP^Sc^ in these tissues [98,99]. Collinge and colleagues re-examined these cases to determine whether Aβ prions could have been transmitted as well as PrP prions. Patients with iCJD originating from human growth hormone contaminated with PrP^Sc^ died at relatively young ages—well before the onset of sporadic AD and the age at which Aβ plaques would accumulate in the brain. In four of the eight brains Collinge examined, severe gray matter loss and vascular Aβ plaque deposition was found [7]. The growth hormone lots responsible for the transmission had detectable Aβ prions [8]. Additional studies demonstrated that injecting these brain fractions into transgenic mice expressing high levels of Aβ caused by fAD mutations accelerated the accumulation of Aβ plaques and CAA, reminiscent of earlier work with PrP transgenic mice. These experiments and those demonstrating transmission of Aβ prions in cell culture strongly support the transmissibility of Aβ prions and emphasize the potential risk of iatrogenic AD and CAA [7,100].

## 10. Tau Prions

Tau is encoded by the *MAPT* gene and is a microtubule-associated protein with six different major tau isoforms [101,102,103,104]. Although the tau that accumulates in AD is invariably wild type, mutations in tau that cause various forms of frontotemporal dementia have helped investigators understand the pathogenesis of the tauopathies using approaches similar to those used on PrP and Aβ prion diseases [105,106]. The majority of clinically relevant mutations are found in an area of the tau protein containing microtubule-binding repeat domains (RDs). The tau protein can contain either three or four RDs. Mutations within an RD may alter the protein’s ability to bind to microtubules, and some mutations increase the likelihood of tau aggregation [107,108].

Similar to PrP prions, tau prions spread in a predictable pattern along neuroanatomical pathways over the course of AD [109] as supported by recent findings from longitudinal studies using tau-PET tracers [110,111,112]. Tau is an intracellular protein but spreads transsynaptically. Among different tauopathies, the localization of tau prions in the brain and the clinical features of disease can vary dramatically. Like PrP [63,65], evidence is accumulating that tau can adopt multiple distinct misfolded conformations, which could lead to separate diseases. Using cultured cells expressing a truncated version of tau fused to a fluorescent protein, Diamond and co-workers showed that tau prions isolated from different tauopathies induced morphologically distinct tau aggregates in the reporter cells [113,114]. A later study in similar cell lines suggested that tau prion propagation required matching isoforms between the prion and its substrate [13]. It is not clear if this process involves mechanisms similar to the species barrier for PrP prions, but results using a line of mice expressing both 3R and 4R tau indicated that transmission of tau prion strains is independent of their isoform composition and depends on strain-specific pathological conformations [115].

Cryo-electron microscopy (cryo-EM) is now being used to decipher the structure of tau prions isolated from human brain samples at the atomic level. Distinct structures of the tau protein are specific to various diseases, including AD [116,117], chronic traumatic encephalopathy (CTE) [118], corticobasal degeneration (CBD) [119], and Pick’s disease [117]. Cryo-EM is having a major effect on the understanding of the defining effects of conformation on prion strains not only for tau prions but also for prions implicated in numerous other NDs.

Goedert and colleagues found that tau prions extracted from the brains of mice expressing a P301S mutant tau transgene induced the aggregation and spread of wild-type human tau following injection into a separate line of mice expressing wild-type tau [76]. Synthetic tau fibrils polymerized in the presence of heparin also induced tau pathology when injected into transgenic mice expressing P301S mutant tau transgenes [120,121]. Tau prions isolated from distinct human tauopathies also induced pathology in mice expressing wild-type human tau, and the morphology was indistinguishable between the induced tau aggregates and the aggregates from the injected tau prions [120]. Onset of pathology occurred much earlier and spread further, however, when the tau prions were inoculated into transgenic mice or reporter cell lines expressing mutant P301S or P301L tau, which appear to be more susceptible to forming prion conformations. The tau prion inocula used in most of these experiments were composed of fibrils or full-length tau bearing P301S or P301L mutations. As discussed for PrP prions, whether the mismatching or host and donor prion sequences and/or conformations produce similar effects is uncertain.

## 11. Alzheimer’s Disease Is a Double Prion Disorder

AD patients accumulate both Aβ and tau inclusions. Many hypotheses attempt to explain the pathogenesis of AD by stressing the relative importance (or lack thereof) of Aβ and tau and how these proteins interact to cause disease. The amyloid cascade hypothesis posits that elevation and aggregation of Aβ initiates a cascade of events, including pathological changes to tau, which is the proximal driver of neurotoxicity [122]. In response to this hypothesis, several in vivo studies in transgenic mice have reported that Aβ is capable of inducing and enhancing the accumulation of tau aggregation [123,124,125,126]. In a recent study, the Aβ and tau prion activities in brain samples from sporadic and inherited AD patients were investigated using a highly sensitive cellular assay [100]. This assay distinguished between biologically active Aβ and tau prions and inactive, inert forms of the proteins. The authors found that Aβ and tau prion activity decreased with age despite an increase in pathological hallmarks such as NFTs and phosphorylated tau. In addition to elucidating a distinction between biologically active and inactive forms of Aβ and tau prions, this study further documented that AD is a double prion disorder.

## 12. α-Synuclein Prions

Genetic linkage studies of inherited forms of PD identified the A53T mutation in the *SNCA* gene, which encodes α-synuclein [127]. Lewy bodies, the hallmark pathology of both sporadic and inherited forms of PD, contain aggregated α-synuclein [128]. Glial cytoplasmic inclusions (GCIs), which are found in the brains of patients with MSA, also contain α-synuclein [78,129,130]. Studies using recombinant α-synuclein, which is a natively unfolded protein and is easily assembled into fibrils when incubated with shaking at high concentrations, showed that the ultrastructural and biochemical properties of the α-synuclein fibrils derived from PD and MSA patients shared many features [131].

Giasson and colleagues created a line of transgenic mice overexpressing the A53T point mutation in α-synuclein that causes inherited PD. Tg(SNCA*A53T)M83^+/−^ (TgM83^+/−^) mice, which are hemizygous for the α-synuclein transgene, do not exhibit signs of neurological dysfunction before 18 months of age, whereas mice homozygous for the transgene (TgM83^+/+^) begin to develop a progressive paralytic phenotype at ~12 months of age [132]. Using the approach pioneered with PrP prions, brain homogenates from ill TgM83^+/+^ mice were injected into healthy TgM83^+/−^ mice. About 180 days after inoculation, the TgM83^+/−^ mice began to show signs of neurological dysfunction and widespread deposits of α-synuclein in their brains [133]. This is very similar to results from PrP studies in which low expressing transgenic rodents were used to rapidly assess prion transmission. Subsequent studies by other groups reported similar results [134]. The next year, our group reported that two human cases of MSA could be transmitted to TgM83^+/−^ mice [14]. Soon thereafter, an additional 14 MSA patient cases were injected into additional TgM83^+/−^ mice to ensure the reproducibility of this remarkable finding. All 14 samples induced disease in these mice in a similar time frame as in the initial experiments; none of the control or PD patient inocula caused neurological dysfunction [135]. Injections of MSA homogenates also induced widespread accumulation of phosphorylated α-synuclein in the brains of symptomatic TgM83^+/−^ mice. In parallel studies, α-synuclein prions from MSA samples induced α-synuclein aggregation in a stable reporter cell line expressing a full-length version of human α-synuclein containing the A53T mutation and fused to yellow fluorescent protein (α-syn140*A53T–YFP) [15,135]. Notably, α-synuclein fibrils injected via peripheral routes also induced α-synuclein neuropathology and disease in this mouse line [136,137].

**Figure 4:**
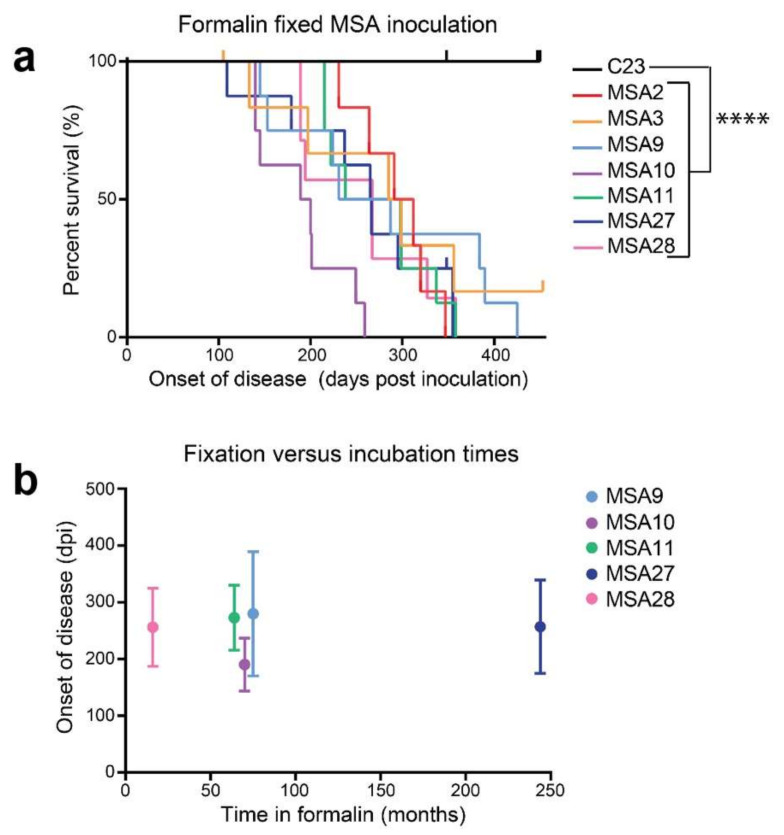
Formalin fixation does not inactivate MSA prions. Formalin-fixed tissue from one control and seven MSA patient samples was homogenized in DPBS, and 30 μL of a 1% homogenate was inoculated intracranially into TgM83^+/−^ mice. (**a**) Kaplan–Meier plot shows onset of neurological signs in TgM83^+/−^ mice following inoculation. Upticks indicate mice that died for reasons other than synucleinopathy. (**b**) Plot comparing the length of time that five of the MSA samples spent in formalin versus the incubation time in TgM83^+/−^ mice. Fixation times ranged from 16 months (MSA28) to 244 months (MSA27), but the infectivity remained constant. Incubation times plotted as mean ± standard deviation. **** *p* < 0.0001. Reprinted with permission from Springer Nature Customer Service Centre GmbH: Springer, Acta Neuropathologica, “MSA prions exhibit remarkable stability and resistance to inactivation,” Woerman, A.L., Kazmi, S.A., Patel, S., Freyman, Y., Oehler, A., Aoyagi, A., Mordes, D.A., Halliday, G.M., Middleton, L.T., Gentleman, S.M., Olson, S.H., Prusiner, S.B. ^©^ Springer-Verlag GmbH Germany 2017 (2018).

As discussed earlier, the unusual resistance of PrP prions to treatments that normally inactivate viruses is a defining feature of PrP prions. Several studies explored how similar inactivation methods that failed to affect PrP prions would affect MSA transmissibility [138]. MSA brain tissue that had been stored in formalin, which is commonly used to inactivate viruses for vaccine production, was inoculated into the brains of TgM83^+/−^ mice. Treatment with formalin, even for 244 months in one case, had no effect on the transmissibility of MSA α-synuclein prions. From these and similar studies, it is clear that MSA is a transmissible neurodegenerative disease involving α-synuclein prions. Like PrP-prions, α-synuclein prions are exceptionally resistant to formalin fixation, as shown in Figure 4.

## 13. The Quest for Human Prion Disease Therapeutics

Increasing evidence argues that prions are responsible for many NDs. Each distinct ND is likely caused by a different protein that folds into a prion that propagates and spreads, leading to a particular disease. As agreement in the field of neurodegeneration grows, more investigators are looking to prion biology to discover effective therapeutics for these illnesses.

To date, no biologic or small molecule has proven effective in altering the course of a human ND. In the case of PrP prion diseases, even quantifiable changes in disease-associated markers in response to treatment have remained elusive. Clinical studies targeting Aβ amyloid as a therapeutic target for AD have shown quantifiable changes in the concentration of Aβ monomers in the cerebrospinal fluid and/or amyloid accumulation in the brain but have not reduced cognitive decline nor prevented NFT formation or neuron loss. Verubecestat, a brain-penetrant small molecule BACE1 inhibitor, may have even accelerated cognitive decline in patients despite its robust effects on AD biomarkers [139], possibly ending one of the most ardently pursued investigational avenues into a therapy for AD. Similarly, studies of seven different Aβ antibodies did not produce significant benefits to patients [140].

## 14. Conclusions

There is a growing consensus among the scientific community that many proteins can misfold and become prions, some of which cause neurodegeneration. Originally limited to the PrP protein in scrapie, kuru, and CJD, the mechanism of prion pathogenesis can now be attributed to such proteins as Aβ, tau, and α-synuclein. The list of prions that cause disease will continue to grow as our understanding of NDs and protein structure expands and our technological tools become more advanced. Despite remarkable progress in understanding prion biology, there is not a single therapy that inhibits prion formation and propagation. After a therapy is developed that successfully treats one disease caused by prions, it is likely that the discovery of therapeutics for other NDs will follow soon.

## Figures and Tables

**Table 1:** Properties of some prions causing neurodegenerative diseases.

Disease	Misfolded Protein in Prions	Familial Mutations	Strains	Aggregate/Amyloid Location	Spread within the Brain	Human-to-Human Transmission	Environmental Cause
Kuru	PrP	No	Probably	Extracellular	Interstitial fluid/cerebrospinal fluid and neural pathways	Cannibalism	No evidence
Creutzfeldt–Jakob disease (CJD)	PrP	Yes; <10% familial CJD, Gerstmann–Sträussler–Scheinker (GSS), fatal familial insomnia (FFI)	Yes	Extracellular	Interstitial fluid/cerebrospinal fluid and neural pathways	Iatrogenic: Dura mater grafts, contaminated cadaver-derived human growth hormone	No evidence
Alzheimer’s disease	Aβ1-40, Aβ1-42(43) amyloid plaques	APP, PSEN1, and PSEN2 mutations elevate Aβ levels	Aβ40/42 ratio	Extracellular	Interstitial fluid/CSF	Contaminated cadaver-derived human growth hormone	Head trauma increases risk
Alzheimer’s disease	3R and 4R tau	No; wild-type tau only	Yes	Intracellular neurofibrillary tangles	Transsynaptic along neural pathways	Unknown	Unknown
Chronic traumatic encephalopathy	3R and 4R tau	No; wild-type tau only	Unknown	Extracellular and intracellular; perivascular	Interstitial fluid/CSF	Unknown	Repeated head trauma
Frontotemporal dementia-tau	4R tau	Yes, in ~20% of patients	Yes	Intracellular neurofibrillary tangles	Transsynaptic along neural pathways	Unknown	Unknown
Parkinson’s disease	α-synuclein	Yes, in ~10% of patients	Yes	Intracellular Lewy bodies	Transsynaptic along neural pathways	Unknown	Herbicide/pesticide exposure increases risk
Multiple system atrophy	α-synuclein	No	Yes	Intracellular	Along neural pathways	Unknown	Unknown

PrP, prion diseases (gray); Alzheimer’s disease, a double prion disease (green); other tauopathies (purple); synucleinopathies (brown). Causative prions: PrP prions (gray); Aβ prions (yellow); tau prions (blue); α-synuclein prions (pink).

## Data Availability

Not applicable.

## Abbreviations

AD	Alzheimer’s disease
APP	Amyloid precursor protein
BSE	Bovine spongiform encephalopathy
CAA	Cerebral amyloid angiopathy
CBD	Corticobasal degeneration
CJD	Creutzfeldt–Jakob disease
Cryo-EM	Cryo-electron microscopy
CTE	Chronic traumatic encephalopathy
DY	Drowsy
fAD	Familial Alzheimer’s disease
FFI	Fatal familial insomnia
FTD	Frontotemporal dementia
GCIs	Glial cytoplasmic inclusions
GSS	Gerstmann–Sträussler–Scheinker
HY	Hyper
iCJD	Iatrogenic Creutzfeldt–Jakob disease
MSA	Multiple system atrophy
NDs	Neurodegenerative diseases
NFTs	Neurofibrillary tangles
PD	Parkinson’s disease
PK	Proteinase K
PrP	Prion protein
RDs	Repeat domains
RML	Rocky Mountain Lab
sFI	Sporadic fatal insomnia
SHaPrP	Syrian hamster prion protein
TME	Transmissible mink encephalopathy
vCJD	Variant Creutzfeldt–Jakob disease
